# Hierarchies of vascular risk: gendered perceptions of arterial and venous disease

**DOI:** 10.3389/fsoc.2026.1923281

**Published:** 2026-10-20

**Authors:** Davide Costa, Raffaele Serra

**Affiliations:** 1 Department of Medical and surgical sciences, Interuniversity Center of Phlebolymphology (CIFL), International Research and Educational Program in Clinical and Experimental Biotechnology, University “Magna Graecia” of Catanzaro, Catanzaro, Italy; 2 Department of Medical and Surgical Sciences, University “Magna Graecia” of Catanzaro, Catanzaro, Italy

**Keywords:** chronic venous disease, gender, illness, peripheral arterial disease, sociology of health, vascular risk

## Abstract

This study explores how patients interpret vascular risk through the intersecting lenses of disease type and gender. Drawing on qualitative interviews with 28 patients in Italy diagnosed with peripheral arterial disease (PAD) or chronic venous disease (CVD), the analysis examines how individuals construct meanings around symptoms, risk, and care. Findings reveal a hierarchy of vascular risk in which arterial disease is perceived as life-threatening and generates vigilance, anxiety, and intensive self-monitoring, whereas venous disease is often normalized and trivialized within everyday life. Gender further shapes these perceptions: men tend to minimize symptoms and delay care, while women demonstrate greater bodily attentiveness and concern. Theoretically, the study advances the concept of a socially constructed hierarchy of vascular risk, showing how culturally differentiated meanings of disease severity intersect with gendered embodiment to shape risk perception, symptom interpretation, and healthcare engagement. By integrating sociological theories of risk, biographical disruption, and gendered embodiment within the study of vascular disease, the analysis extends risk scholarship to an underexplored clinical domain and demonstrates how biomedical risk is translated into socially differentiated lived experience.

## 1 Introduction

The ways in which people interpret, respond to, and live with health threats cannot be fully understood solely through objective medical knowledge or biological risk profiles (Zinn, 2005). Risk is socially constructed, negotiated through personal experience and cultural frameworks, and embedded in social contexts where individuals make sense of their bodies and lives (Kavanagh and Broom, 1998). Sociological approaches emphasize the gap between expert definitions of hazard and lay understandings of vulnerability, showing how illness meanings are shaped by social norms, power relations, and everyday practices (Ford et al., 2024). Beck (2009) highlights perceived dangers in modern “risk society,” while Douglas's cultural theory stresses collective meaning making in defining threats (Rippl, 2002). Despite research on cancer, diabetes, or infectious diseases, vascular conditions, including arterial and venous diseases, remain understudied sociologically (Costa and Serra, 2026a).

Peripheral artery disease (PAD) is a circulatory condition in which narrowed or blocked arteries reduce blood flow to the limbs, most commonly the legs. It is usually caused by atherosclerosis (buildup of fatty plaques in the arteries). PAD can lead to symptoms such as leg pain during walking (intermittent claudication), and in severe cases, poor wound healing, tissue damage, or chronick limb- ischemia (CLTI). PAD prevalence differs by sex, race, and ethnicity, contributing to variations in disease burden. Globally, PAD affects an estimated 200 million people. Prevalence estimates depend on diagnostic criteria, with studies limited to symptomatic cases often underestimating the true burden. Rates increase significantly with age, peaking in individuals over 70 years. Severe PAD, including CLTI, which may lead to amputation, affects about 1.3% of adults in the United States. When defined by an ankle-brachial index (ABI) ≤ 0.9, PAD prevalence is generally similar between men and women (around 3%−4.5% in those aged ≥40), although some studies report slightly higher rates in women (3.5% vs. 2.5% in men). In contrast, symptomatic PAD, such as intermittent claudication, is more commonly reported in men. Globally, PAD is more prevalent in women, particularly after midlife, while men tend to experience greater symptom burden, higher mortality, and more severe disease outcomes (GBD 2019 Peripheral Artery Disease Collaborators, 2023; Fu and Zhang, 2025).

Chronic venous disease (CVD) is a condition in which veins, most commonly in the legs, are unable to efficiently return blood to the heart, often due to valve dysfunction or weakened vein walls. It can lead to symptoms such as leg swelling, heaviness, aching, varicose veins, skin changes, and, in advanced cases, venous ulcers. Prevalence estimates vary by region, with varicose veins reported in up to 57% of men and 77% of women worldwide (Serra et al., 2016).

Unlike PAD, individuals with CVD often perceive their symptom such as swelling, discomfort, or varicosities, as minor, cosmetic, or simply part of aging, despite evidence that CVD can significantly affect function and quality of life. This contrast highlights how social perceptions shape the understanding of vascular disease, suggesting that the perceived severity of illness is influenced not only by clinical measures but also by social and cultural interpretations. Gender further complicates these processes of interpretation. Research in sociology of health and illness has shown that norms of femininity and masculinity influence how individuals attend to bodily sensations, articulate symptoms, and engage with health care (Courtenay, 2000). Masculine ideals that valorise stoicism and self-sufficiency can encourage minimization of symptoms and delayed help-seeking, while women's health narratives often reflect greater attentiveness to bodily changes and proactive engagement with medical care (Moore et al., 2013). Yet, despite growing recognition of these gendered patterns in illness experience, there remains a gap in understanding how gender intersects with differing constructions of risk across disease categories, particularly in the domain of chronic vascular conditions (Regitz-Zagrosek and Gebhard, 2023).

The study contributes to a nuanced understanding of the social life of health risks, showing that vascular disease is not merely biomedical but a lived experience to be interpreted and negotiated within cultural and social contexts. It emphasizes the need for health communication and clinical practice to recognize that patients actively construct their own frameworks of meaning, shaped by disease characteristics, gendered expectations, and social positioning. We examine patients' perceptions of vascular risk comparatively by disease type and gender, asking how those with arterial or venous conditions interpret risk in daily life and interactions with healthcare services. Using qualitative interviews with patients diagnosed with PAD or CVD in Italy, we draw on medical sociology scholarship on risk perception, embodiment, and gender to situate experiences within broader social contexts and explore how gender influences symptom attention, bodily interpretation, and care engagement.

## 2 Theoretical framework

### 2.1 Risk society and the social construction of vascular risk

Understanding how patients interpret and respond to vascular conditions requires situating perceptions with in sociological theories of risk. Beck (1996, 2009, 2014) concept of the risk society provides a framework for analyzing how societies organize knowledge, anxiety, and decision-making around threats. Risk is socially mediated, shaped by institutions, expert knowledge, and public discourse (Burrows et al., 1995), and individuals constantly interpret and respond to hazards affecting their bodies and futures (Nettleton, 2021). Manufactured risks (Beck, 2008, 2009) emerge from technological, organizational, and institutional processes, including those operating within healthcare systems, where diagnostic practices, treatment pathways, risk communication, and the organization of care contribute to defining which health threats are recognized as urgent and how patients are expected to respond to them (Michaels and Monforton, 2005). Vascular disease exemplifies this process: arterial conditions are commonly framed as urgent threats requiring close surveillance and preventive action, whereas venous disorders are more often represented as chronic and comparatively benign (Costa and Serra, 2026a,b), reflecting broader social and institutional processes that shape the cultural salience of different forms of disease (Williams, 2006). Patients interpret risk through interactions between clinical knowledge, cultural narratives, and healthcare organization (Nettleton, 2021), assuming responsibility for monitoring, prevention, and treatment (Beck, 2008; Nettleton, 2020), with perception varying by whether the condition seems life-threatening or manageable (Costa and Serra, 2026a).

### 2.2 Cultural risk hierarchies and biographical disruption

While Beck highlights societal organization of risk, Douglas (2002) cultural theory, shows how societies symbolically classify dangers, structuring perceptions through cultural systems that determine which threats are serious or normalized (Lupton, 1993). Applied to vascular health, this explains implicit hierarchies of risk (Sol et al., 2009): arterial disease, linked to acute outcomes like myocardial infarction, stroke, or amputation, is seen as urgent (Arnold et al., 2023), whereas venous disease is framed as common and non-threatening despite discomfort and mobility issues (Ballard and Bergan, 2000). Patients' interpretations emerge within these hierarchies (Wong and King, 2008), influencing care, symptom monitoring, and routines (Gibson and Kenrick, 1998; Kleinman et al., 1978). Biographical disruption (Bury, 1982, 2001) highlights how chronic illness interrupts life continuity. Arterial disease increases vulnerability and bodily vigilance (Campia et al., 2019), while venous disease is often normalized through manageable strategies (Franks et al., 2016; Walker and Rogers, 2017).

Moreover, current research also suggests that responses to chronic disease risk cannot be understood solely in terms of the objective probability or severity of adverse outcomes. Individuals actively interpret, prioritize, or sometimes avoid health-risk information according to their existing perceptions of health, anticipated emotional consequences, and the perceived usefulness of such information. Risk perception in chronic illness should therefore be understood as a dynamic interpretive process that mediates the relationship between biomedical information and everyday health behavior (Wang et al., 2026).

### 2.3 Gendered embodiment and narratives of vascular risk

Perceptions of illness and risk are shaped by gendered social expectations regarding the body, vulnerability, and help-seeking (Kang et al., 2025). Sociological research shows illness is mediated through embodiment, where social norms are inscribed in bodily practices (Freund, 1990). Gendered embodiment affects how men and women attend to and interpret bodily sensations (del Río Carral and Lyons, 2020). Masculine norms of stoicism may lead men to downplay CVD symptoms (Costa et al., 2023), while women's vigilance toward bodily change, particularly in venous disease, reflects caregiving, aesthetic, and health expectations (Van Manen, 1998; Costa et al., 2023). These gendered dynamics interact with cultural hierarchies of vascular risk, shaping symptom interpretation, anxiety, and care-seeking (Costa et al., 2023). Integrating risk society, cultural risk, biographical disruption, and gendered embodiment frameworks, the study treats vascular risk perception as socially situated, influenced by hierarchies of danger, embodied experiences, and gendered expectations.

In this context, Recent qualitative research further demonstrates that chronic illness produces forms of embodied knowledge through which individuals learn to interpret bodily changes, anticipate symptoms, and adapt self-management practices over time. Importantly, such embodied knowledge is not socially neutral but may intersect with gendered experiences and identities, reinforcing the need to understand chronic disease management as both a bodily and socially situated process (Sufian et al., 2024).

## 3 Methods

This qualitative study builds on semi-structured interviews conducted with 28 patients diagnosed with either PAD or CVD. These interviews were designed to explore patients' perceptions of vascular risk, emotional responses to their condition, and the ways in which gendered expectations shaped their experiences. The research protocol received approval from the Institutional Review Board of the Interuniversity Center of Phlebolymphology (CIFL), within the framework of the International Research and Educational Program in Clinical and Experimental Biotechnology (ER.ALL.2018.77). Prior to participating in the interviews, all patients and their parents or legal guardians provided written informed consent. Participants were clearly informed that both the content of the interviews and the audio recordings would remain confidential and anonymous. All procedures involving human participants were conducted in compliance with the ethical principles outlined in the Declaration of Helsinki.

Between 2023 and 2025, participants were recruited through vascular clinic in Calabria, South Italy. The invitation to participate explained that the interviews would focus broadly on patients' experiences of living with vascular disease, including their interpretation of symptoms, perception of risk, engagement with healthcare services, and emotional responses. Inclusion criteria required participants to be aged 18 or older, have a confirmed diagnosis of either arterial or venous disease for at least 6 months, and be able to communicate in Italian. A total of 39 individuals expressed interest, and 28 ultimately participated.

Each participant was interviewed individually by one of the researchers. Interviews were conducted primarily in participants' homes or in quiet spaces within healthcare facilities and lasted on average 40 to 50 min. Two interviews were conducted digitally due to participants' geographic constraints. A semi-structured interview guide was used to organize the interviews while allowing participants to elaborate freely on issues they considered personally significant. The guide covered perceptions of vascular risk, bodily awareness, symptom interpretation and management, emotional responses, healthcare engagement, normalization of symptoms, social context, and the possible influence of gender on illness experience. Questions were formulated in an open-ended and non-directive manner, with optional prompts used only to facilitate clarification or further elaboration. Gender-related questions were introduced toward the later stages of the interview, after participants had first described their experiences in their own terms, in order to minimize potential interviewer prompting or framing. The main interview domains, questions, and optional prompts are presented in Table 1. All interviews were audio-recorded, transcribed verbatim, and anonymized through pseudonyms.

**Table 1:** Semi-structured interview guide: domains, principal questions, and optional prompts.

Domain	Principal open-ended questions	Optional prompts
**Experience of vascular disease**	Could you tell me about your experience of living with your vascular condition? What does having this condition mean to you in everyday life?	First symptoms; changes over time; effects on daily activities, work, family life, mobility, and social activities.
**Symptom interpretation and bodily awareness**	How do you usually understand or interpret the symptoms associated with your condition? How much attention do you pay to bodily changes related to your condition?	Symptoms considered important; monitoring of legs, feet, skin, swelling, or temperature; changes considered significant; changes in symptom interpretation after diagnosis.
**Perception of risk and disease severity**	How serious do you consider your vascular condition to be? When you think about your condition, what kinds of future health problems, if any, come to mind?	Perceived complications; perceived likelihood of complications; influence of information received from healthcare professionals; comparison with other illnesses.
**Emotional responses and anticipation**	How does your vascular condition make you feel? Do you find yourself thinking about what might happen to your health in the future because of this condition?	Worry, fear, frustration, uncertainty; situations increasing concern; anticipation of deterioration; influence on daily decisions and bodily monitoring.
**Everyday management and self-care**	What do you usually do to manage your vascular condition in everyday life? How do you decide whether a symptom can be managed yourself or requires medical attention?	Medication; compression therapy; exercise; diet; rest or leg elevation; adherence to recommendations; reasons for seeking or postponing medical advice.
**Healthcare engagement and communication**	Could you describe your experiences of discussing your vascular condition with healthcare professionals? What influences your decision to seek medical care for new or worsening symptoms?	Understanding of medical information; communication of risk; ability to discuss concerns; previous healthcare experiences; family influence; practical barriers to care.
**Normalization and everyday explanations**	Are there aspects of your condition that you have come to regard as a normal part of everyday life?	Symptoms participants have learned to live with; aging; occupational strain; lifestyle; family history; influence of normalization on healthcare-seeking.
**Gender and illness experience**	In your experience, do you think being a man or a woman has any influence on how people experience or respond to health problems such as yours? Do you think your own response to your vascular condition has been influenced in any way by gender-related expectations?	Participants could explicitly report no gender influence; bodily attention; emotional expression; help-seeking; self-management; independence; family/caregiving roles; appearance and social expectations.
**Social context and individual variation**	What other aspects of your life influence the way you experience and manage your condition? Do you know people with a similar condition who respond differently from you?	Age; work; family responsibilities; social support; financial or practical circumstances; previous illness experiences; possible explanations for differences between individuals.
**Closing/reflexive questions**	Is there anything about living with your vascular condition that you think is important but that we have not discussed? What would you want healthcare professionals to understand about patients' experiences of this condition?	Any issues not captured by the preceding domains; participant-defined priorities and concerns.

The interview data were analyzed using reflexive thematic analysis following Clarke and Braun (2017) six-phase approach. Initial familiarization with the transcripts was followed by systematic coding to identify recurring patterns related to risk perception, emotional responses, and gendered experiences. Initial coding, conducted by D.C., generated approximately 60 descriptive and interpretive codes addressing symptom interpretation, perceived disease severity, bodily monitoring, emotional responses, normalization of symptoms, healthcare-seeking behavior, self-management practices, and gendered understandings of illness. Through iterative comparison and conceptual refinement, overlapping codes were consolidated into broader categories, which were subsequently organized into four overarching themes: hierarchies of vascular risk, embodied anxiety and anticipation, trivialization and normalization of venous disease, and gendered interpretations. Consistent with the principles of reflexive thematic analysis, coding was treated as an interpretive and reflexive process rather than as an exercise requiring statistical inter-coder reliability. The developing thematic structure was then considered in relation to the empirical material and the theoretical literature on risk society, cultural constructions of health, and gendered embodiment. Special attention was given to contrasting narratives of arterial and venous patients: arterial patients frequently described heightened vigilance and anxiety, whereas venous patients often downplayed or normalized symptoms. Gendered dimensions emerged across both patient groups, shaping how bodily symptoms were monitored, interpreted, and communicated. Themes were refined through an abductive process, linking patterns in the data to theoretical constructs of risk perception, biographical disruption, and gendered embodiment. Thematic sufficiency was considered to have been reached when successive interviews no longer generated substantively new patterns relevant to the research questions and the developing thematic framework provided adequate conceptual coverage of participants‘ accounts across both disease groups and genders (Braun and Clarke, 2025). Recruitment and analysis proceeded iteratively, allowing emerging patterns to be assessed against subsequent interviews. Toward the final interviews, new data primarily elaborated or provided variations on already identified patterns rather than generating additional major themes. This approach allowed us to examine how patients construct hierarchies of vascular risk, manage emotional responses, and navigate healthcare systems within the social and cultural context of Italy. The final analysis yielded four overarching themes, each capturing a distinct aspect of patients' lived experience: the hierarchy of vascular risk (how arterial disease is experienced as more threatening than venous disease), embodied vigilance and anxiety among arterial patients, and normalization and minimization of venous disease. Gendered patterns were integrated within each theme to highlight differences in perception, attention to bodily signals, and engagement with healthcare.

The study is situated in the Italian healthcare context, characterized by a nationally regulated but regionally administered system. Patients' experiences are shaped by access to specialist vascular care, local variations in service provision, and evolving patient expectations. Understanding these experiences requires considering both the structural dimensions of healthcare delivery and the interpretive work that patients engage in as they make sense of risk, manage symptoms, and negotiate their own care trajectories.

The final sample comprised 28 participants, including 14 men and 14 women, aged 42–78 years. Fourteen participants had a diagnosis of PAD and 14 had CVD. All participants had been living with their vascular condition for at least 6 months, in accordance with the study inclusion criteria. Participant characteristics are summarized in Table 2. Pseudonyms are used throughout the manuscript to protect participants' identities.

**Table 2:** Sociodemographic and clinical characteristics of participants.

Pseudonym	Age (years)	Sex	Diagnosis	Occupation	Duration since diagnosis
Paolo	62	Male	PAD	Construction worker	4 years
Carla	57	Female	PAD	Administrative employee	3 years
Luigi	59	Male	PAD	Mechanic	5 years
Alessia	54	Female	PAD	Teacher	2 years
Luca	60	Male	PAD	Shopkeeper	4 years
Elena	58	Female	PAD	Nurse	3 years
Antonio	71	Male	PAD	Retired factory worker	8 years
Francesco	66	Male	PAD	Farmer	6 years
Giuseppe	73	Male	PAD	Retired construction worker	7 years
Maria	64	Female	PAD	Homemaker	5 years
Rosa	69	Female	PAD	Retired administrative employee	6 years
Giovanni	78	Male	PAD	Retired agricultural worker	10 years
Teresa	67	Female	PAD	Retired teacher	4 years
Andrea	52	Male	PAD	Self-employed worker	2 years
Giulia	65	Female	CVD	Homemaker	9 years
Marco	70	Male	CVD	Retired manual worker	12 years
Sara	61	Female	CVD	Shop assistant	6 years
Roberto	67	Male	CVD	Retired technician	10 years
Lucia	62	Female	CVD	Retail worker	4 years
Anna	63	Female	CVD	Administrative employee	7 years
Matteo	68	Male	CVD	Retired craftsman	8 years
Francesca	49	Female	CVD	Office employee	3 years
Stefano	56	Male	CVD	Driver	5 years
Daniela	55	Female	CVD	Teacher	4 years
Salvatore	74	Male	CVD	Retired agricultural worker	15 years
Caterina	72	Female	CVD	Retired shopkeeper	11 years
Pietro	76	Male	CVD	Retired factory worker	13 years
Laura	42	Female	CVD	Healthcare worker	2 years

## 4 Results

In our analysis, we identified four thematic patterns in how patients perceive and respond to vascular risk. These themes illustrate how risk perception is closely intertwined with patients' daily lives, identities, and experiences within the healthcare system, and how these perceptions are shaped by both disease type and gender.

### 4.1 Hierarchies of vascular risk

Participants' accounts revealed a clear hierarchy in the perceived seriousness of vascular conditions. This hierarchy was primarily organized around disease type and the anticipated severity of its consequences. Arterial disease was generally positioned at the upper end of this hierarchy, being associated with potentially catastrophic outcomes such as myocardial infarction, stroke, limb loss, and sudden deterioration. CVD, by contrast, was typically positioned lower in the hierarchy and described as uncomfortable or disruptive but comparatively less threatening to life.

This distinction was evident in the language participants used to describe their conditions. Paolo, a 62-year-old participant with PAD, captured the perceived immediacy of arterial risk:

“Every twinge in my leg makes me think, could it be a blockage? I try to stay calm, but it's always there in the back of my mind.”

In contrast, Giulia, a 65-year-old participant with venous insufficiency, described her condition in markedly less threatening terms:

“My legs hurt sometimes, especially at the end of the day, but I just put on my stockings and keep going. […] It's not like it could kill me, so I don't worry too much.”

The contrast between these accounts illustrates that participants did not evaluate vascular conditions solely through the presence or intensity of physical symptoms. Instead, perceived seriousness was strongly related to what participants believed the disease could ultimately cause. Arterial disease acquired meaning through its association with potentially irreversible or life-threatening outcomes, whereas venous disease was more readily situated within the domain of chronic discomfort and everyday management.

Social comparison also contributed to this hierarchy. Participants with venous disease frequently evaluated their condition relative to illnesses they regarded as more serious, including cardiovascular disease and cancer. Such comparisons reduced the perceived urgency of venous symptoms and situated CVD within a broader social classification of illness severity. Thus, biomedical knowledge interacted with personal experience, observations of other people's illnesses, and culturally shared understandings of what constitutes a serious disease.

These accounts support the concept of a socially constructed hierarchy of vascular risk.

The distinction between arterial and venous disease was not simply a reflection of clinical classifications but an interpretive framework through which participants evaluated vulnerability, seriousness, and the significance of their symptoms. The consequences of this hierarchy for embodied vigilance and anticipatory anxiety, the normalization of venous disease, and gendered interpretations of symptoms and healthcare engagement are examined separately in the following sections.

### 4.2 Embodied anxiety and anticipation

For patients living with arterial disease, perceptions of vascular risk were not only cognitive evaluations of potential danger but also deeply embodied experiences that shaped how they related to their own bodies in everyday life. Participants frequently described developing a heightened sensitivity to bodily sensations, paying close attention to subtle physical changes that might signal worsening circulation or the onset of serious complications. Tingling, numbness, fatigue, or changes in skin temperature were often interpreted as potential warning signs, requiring careful observation and evaluation.

Luigi, a 59-year-old man diagnosed with PAD, described how his daily routine had gradually become organized around this form of bodily monitoring:

“Every morning I check my legs. It has become almost automatic now. I look at the color of the skin, I touch them to see if they feel cold, and sometimes I press on my feet to see if there is any swelling. Even a small ache can make me nervous. I start wondering if the circulation is getting worse or if something is blocked somewhere. It's not that I want to think like that, but it's difficult to ignore the signals when you know what could happen.”

Luigi's narrative illustrates how the body becomes a site of continuous observation, requiring ongoing interpretive work. What might once have been perceived as ordinary bodily sensations are now imbued with diagnostic significance. In sociological terms, this reflects the form of “illness work” described by Corbin and Strauss (1985), in which patients must actively monitor and manage their physical condition as part of everyday life with chronic disease. The body is no longer taken for granted but instead becomes an object of scrutiny, where signs and sensations are interpreted through the lens of potential risk.

Several participants described how this heightened bodily awareness produced a persistent state of anticipatory anxiety. Rather than responding only to immediate symptoms, patients often found themselves imagining possible future complications. Alessia, a 54-year-old woman with arterial disease, explained how these anticipatory concerns shaped her emotional experience:

“Sometimes I wake up in the middle of the night and start checking my pulse or touching my feet to make sure everything feels normal. It's not that something has necessarily happened, but my mind goes straight to the worst possibilities. I imagine the circulation getting worse or something happening suddenly. It's exhausting because you know you should relax, but you also feel that you have to pay attention.”

Alessia's account highlights the temporal dimension of living with vascular risk. The body is experienced not only in its present condition but also as a site of potential future deterioration. Patients become oriented toward the anticipation of complications, constantly evaluating whether current sensations might indicate the beginning of a more serious event. This anticipatory orientation reflects what Bury (1982) described as the disruption of the taken-for-granted body in chronic illness. When illness enters the biography, bodily sensations that were previously ignored acquire new significance, demanding interpretation and management.

For many arterial patients, this process resulted in a continuous negotiation between vigilance and anxiety. On the one hand, patients recognized the importance of monitoring symptoms and adhering to medical recommendations. On the other hand, the constant attention required by this monitoring could itself become psychologically burdensome. Some participants described feeling trapped between the need to remain alert and the desire to maintain a sense of normalcy in everyday life.

The body, therefore, becomes both a source of information and a source of uncertainty. Patients described learning to interpret bodily signals through experience, often drawing on previous episodes of illness or medical consultations to determine which sensations required concern. This interpretive process illustrates the complex forms of knowledge patients develop as they learn to live with chronic vascular conditions.

In contrast, patients living with CVD often described a markedly different relationship with their bodies. While they were aware of symptoms such as swelling, heaviness, or visible varicose veins, these sensations were rarely interpreted as indicators of serious medical risk. Instead, they were often framed as familiar and manageable aspects of everyday bodily experience.

Sara, a 61-year-old woman diagnosed with venous insufficiency, explained how she approached these symptoms:

“Sometimes my legs swell after a long day, especially if I've been standing a lot. But for me it's just something I've learned to live with. I wear my compression stockings, and that usually helps. What bothers me more is when my legs feel heavy or when I can't wear certain shoes comfortably. I don't really think about serious consequences.”

Sara's narrative suggests a different form of embodied attention, one oriented less toward the anticipation of medical danger and more toward maintaining comfort and everyday functionality. Rather than interpreting bodily sensations as warning signs of catastrophic events, venous patients often treated them as manageable inconveniences requiring practical solutions.

Roberto, a 67-year-old man with long-standing varicose veins, described a similar perspective:

“I've had these veins for many years. Sometimes they ache, especially if I've been walking a lot or standing too long. But it's mostly annoying rather than worrying. I just sit down for a while, raise my legs, and it passes. For me it's something you learn to deal with.”

Roberto's account illustrates how venous disease becomes integrated into daily life through practical routines and coping strategies. Rather than prompting constant surveillance of bodily signals, the condition is incorporated into ordinary patterns of activity and rest.

These contrasting narratives highlight how embodied experiences of vascular disease are shaped not only by physiological processes but also by the meanings patients attribute to different types of symptoms. For arterial patients, bodily sensations are interpreted within a framework of high-risk awareness, producing vigilance and anticipatory anxiety. For venous patients, similar sensations are more likely to be normalized, understood as manageable features of everyday life rather than signals of imminent danger.

Gender further shaped these embodied responses. Women with arterial disease frequently described a heightened attentiveness to bodily changes, often articulating their vigilance in emotional and relational terms. Their narratives emphasized concern about losing mobility, independence, or the ability to care for family members. This attentiveness was sometimes accompanied by expressions of worry or fear regarding possible complications.

Men, by contrast, often described their monitoring practices in more restrained terms. While they reported engaging in careful observation of symptoms, their narratives tended to emphasize control, discipline, and responsibility rather than anxiety. This pattern reflects broader sociological observations about gendered norms of emotional expression and bodily awareness (Connell, 2005; Butler, 1999).

Among venous patients, gendered differences also emerged in how bodily attention was directed. Women were more likely to monitor visible changes in the legs, paying attention to swelling, skin appearance, or the development of new varicose veins. These concerns were connected not only to aesthetics but also to comfort and physical wellbeing. Men, in contrast, often minimized bodily discomfort and focused primarily on maintaining everyday routines, interpreting symptoms as minor inconveniences rather than problems requiring close attention.

These findings demonstrate that embodied anxiety and anticipation are closely linked to both disease type and gendered patterns of bodily awareness. Arterial patients frequently experience their bodies as fragile and potentially unstable, requiring continuous monitoring and interpretation. Venous patients, by contrast, often develop practical coping strategies that allow them to incorporate symptoms into the rhythms of everyday life without extensive concern for serious consequences.

These contrasting experiences illustrate how physical sensations and emotional responses become intertwined in shaping patients' relationships with their bodies. The experience of vascular illness is therefore not limited to clinical symptoms but also involves ongoing interpretive work through which patients attempt to make sense of their bodily sensations and anticipate possible futures.

### 4.3 Trivialisation and normalization of venous disease

Patients living with CVD frequently described their condition in ways that minimized its perceived seriousness. Rather than framing symptoms as indicators of medical risk, participants often interpreted swelling, discomfort, and visible varicosities as ordinary bodily changes associated with aging, occupational strain, or family predisposition. This process of trivialization shaped how patients understood their condition and, importantly, how they engaged with healthcare services.

For many participants, venous disease was not initially recognized as a medical issue requiring professional attention. Instead, symptoms were interpreted within familiar social narratives about tired legs, long working hours, or hereditary traits. Caterina, a 72-year-old woman diagnosed with venous insufficiency, explained how she had gradually come to view her symptoms as part of normal bodily experience:

“My legs swell sometimes, especially at the end of the day, but it doesn't really worry me. My mother had the same problem, and my sisters too. In our family it's something quite common. I just put on my stockings and try to rest when I can. It's uncomfortable sometimes, but it's not something I see as serious.”

Caterina's account illustrates a form of normalization in which the presence of symptoms is interpreted through familial and social comparisons. By situating her condition within a broader pattern shared by relatives, the symptoms lose their sense of urgency and instead become incorporated into an ordinary narrative of bodily variation. This kind of interpretive strategy allows patients to maintain continuity in their everyday lives without redefining themselves as seriously ill.

Marco, a 70-year-old man with long-standing varicose veins, described a similar approach to his condition:

“Sure, the veins look bad, and sometimes they ache a bit, but I've had them for many years now. It's something you get used to. When you're younger you might worry about things like that, but at my age it feels more like part of getting older. I know people with heart problems or cancer, so compared to that it doesn't feel like a big issue.”

Marco's narrative reveals how patients frequently evaluate the seriousness of their illness through social comparison. Rather than interpreting symptoms exclusively through biomedical knowledge, participants positioned their condition within a broader hierarchy of illness severity. By comparing venous disease with conditions perceived as more dangerous or life-threatening, such as cardiovascular disease or cancer, participants often framed their symptoms as relatively minor.

This process reflects what sociologists of health have described as normalization strategies, through which individuals reinterpret symptoms in ways that preserve a sense of normal life (Bury, 1982; Corbin and Strauss, 1985). Instead of allowing the condition to disrupt everyday routines, patients integrate symptoms into their daily activities, adapting through practical coping mechanisms rather than seeking immediate medical intervention.

However, this normalization often had consequences for health-seeking behavior. Several participants explained that they delayed consulting healthcare professionals because they believed their symptoms were ordinary or inevitable. Lucia, a 62-year-old woman, described how she postponed medical evaluation for a considerable period:

“At first I didn't think about going to the doctor. My legs were swollen sometimes, but I thought it was because I stand all day at work. Many of my colleagues have the same problem, so it didn't seem unusual. It was only when the swelling became much worse that I started to worry and decided to get it checked.”

Lucia's account highlights how everyday explanations can postpone medical attention. By attributing her symptoms to occupational fatigue rather than vascular disease, she was able to maintain a sense of normality for a long period of time. Only when the condition progressed to a point where it could no longer be easily explained within these familiar narratives did she seek professional care.

Participants frequently described similar patterns of delayed engagement with healthcare services. The normalization of symptoms allowed them to continue their daily routines, but it also meant that early signs of venous complications could go unnoticed or unaddressed. As a result, some patients only sought medical advice once symptoms had intensified significantly.

The trivialization of venous disease was also reinforced through broader social discourses about aging and bodily wear. Many participants framed symptoms such as swelling or visible veins as natural consequences of growing older, rather than as signs of a medical condition requiring intervention. These interpretations reflected widely shared cultural expectations about the aging body, where discomfort and physical changes are often considered inevitable.

Gender further shaped these patterns of interpretation. Women frequently reported paying attention to the visible and aesthetic aspects of venous disease, including swelling, skin changes, and the appearance of varicose veins. While these concerns were sometimes framed in terms of physical discomfort, they were also connected to issues of bodily appearance and social presentation. Anna, a 63-year-old woman living with venous disease, described how these changes affected her everyday life:

“I notice the swelling especially when I want to wear certain clothes or shoes. Sometimes my legs feel heavy, and I try to rest or elevate them. I wear compression stockings because they help, but I also think about how my legs look, especially in the summer.”

Anna's narrative illustrates how bodily attention among women often included both functional and aesthetic considerations. Monitoring symptoms was not only about managing discomfort but also about maintaining a sense of bodily control and social confidence.

Men, by contrast, were generally more likely to dismiss venous symptoms as insignificant. Rather than closely monitoring swelling or discomfort, many male participants described adopting a more dismissive attitude toward their condition. They often interpreted symptoms as ordinary signs of aging or occupational strain, and consequently saw little reason to seek medical advice unless the problem became severe.

This gendered pattern reflects broader sociological observations regarding health behavior and masculine norms. Cultural expectations surrounding masculinity often encourage men to minimize bodily concerns and delay seeking care, particularly for conditions perceived as non-life-threatening (Connell, 2005). Within the context of venous disease, this tendency further reinforced the trivialization of symptoms.

These narratives demonstrate that the trivialization and normalization of venous disease are not merely individual attitudes but socially structured processes. Patients interpret their symptoms through familial experiences, cultural narratives about aging, and comparisons with other illnesses. These interpretive frameworks allow them to maintain continuity in their everyday lives but may also contribute to delayed diagnosis and limited engagement with preventive care.

The contrast with arterial disease becomes particularly striking in this context. While arterial patients often experience heightened vigilance and anxiety due to the perceived severity of potential complications, venous patients frequently adopt normalization strategies that reduce the perceived urgency of their condition. These differences reveal how perceptions of vascular risk are shaped not only by medical knowledge but also by the social meanings patients attribute to different forms of illness.

Understanding these processes is essential for interpreting patient behavior within vascular care pathways. When venous disease is framed as an ordinary part of aging or daily life, patients may delay seeking treatment until symptoms become more severe. In this way, normalization strategies that help maintain a sense of normalcy can simultaneously obscure the potential progression of the condition, illustrating the complex relationship between social interpretations of illness and patterns of healthcare utilization.

### 4.4 Gendered interpretations

Gender played a central role in shaping how patients perceived, interpreted, and responded to vascular risk. While the type of vascular condition strongly influenced patients' perceptions of severity and vulnerability, gendered expectations about bodily awareness, emotional expression, and help-seeking behavior intersected with these perceptions in important ways. Patients' narratives suggested that men and women often approached symptoms, risk, and medical care differently, reflecting broader social norms regarding health, responsibility, and bodily attention.

Among men, both those living with arterial disease and those with venous conditions often described a tendency to minimize symptoms or delay seeking medical advice. Many male participants framed their approach to illness through ideals of self-reliance and endurance, emphasizing the importance of continuing daily activities despite physical discomfort. Luca, a 60-year-old man living with arterial disease, described how he approached his diagnosis:

“I know my arteries are blocked, and of course the doctors told me to be careful. But I try not to think about it too much. If you keep worrying about every little thing you can't live your life. I go to the doctor when I have to, but otherwise I try to handle things myself.”

Luca's account illustrates a pattern frequently observed in the interviews, where men acknowledged the seriousness of their condition but simultaneously attempted to limit the emotional impact of the diagnosis. Rather than openly expressing anxiety, they often framed their response in terms of personal discipline and control. This form of stoic vigilance allowed them to maintain a sense of autonomy while still engaging with preventive practices such as medication adherence or lifestyle changes.

Sociological analyses of masculinity have long highlighted the ways in which health behaviors are shaped by cultural expectations surrounding strength, independence, and emotional restraint (Connell, 2005). Within this framework, acknowledging vulnerability may be perceived as inconsistent with dominant masculine ideals. As a result, men may adopt strategies that allow them to maintain a sense of resilience while minimizing overt expressions of concern.

Matteo, a 68-year-old man with venous insufficiency, described a similar approach when discussing his symptoms:

“The veins hurt a little sometimes, especially after walking a lot, but it's nothing serious. Other men in my family have the same problem. It's something that comes with age. I'll go to the doctor if it gets really bad, but otherwise you just carry on.”

Matteo's narrative reflects the normalization strategies often observed among male venous patients. Symptoms were framed as ordinary bodily experiences rather than medical issues requiring close attention. By interpreting discomfort as a natural part of aging or family predisposition, patients were able to maintain continuity in their daily lives without redefining themselves as ill.

In contrast, women frequently described a more attentive relationship with their bodies and symptoms. Female participants often reported closely monitoring bodily changes, expressing concern not only about potential medical consequences but also about the broader impact of illness on everyday functioning and social life. Elena, a 58-year-old woman living with arterial disease, described the vigilance she developed after receiving her diagnosis:

“I check my legs constantly because I'm afraid of missing something important. If the circulation changes or if I notice something unusual, I want to know immediately. The idea that something serious could happen makes me very careful. I want to do everything possible to prevent it.”

Elena's narrative illustrates a form of attentive monitoring that combines vigilance with emotional awareness. Unlike many male participants who emphasized stoic control, women often articulated their vigilance through expressions of concern, worry, or responsibility toward their own health. These accounts suggest a heightened sensitivity to bodily signals, accompanied by a strong motivation to prevent complications.

Women with venous disease also described monitoring their bodies, though their concerns were often oriented toward comfort and bodily appearance rather than catastrophic medical outcomes. Daniela, a 55-year-old woman living with venous disease, explained how she paid attention to the visible and physical changes associated with her condition:

“I notice when my legs start swelling or when the veins look more visible. It bothers me because they feel heavy and sometimes they don't look very nice either. I wear compression stockings and try to take care of them as much as I can.”

Daniela's narrative highlights how bodily attention among venous patients frequently includes both functional and aesthetic considerations. While the condition was rarely perceived as life-threatening, the physical changes associated with venous disease could still influence everyday comfort and self-perception. Monitoring symptoms therefore became part of maintaining both physical wellbeing and social confidence.

The intersection between disease type and gender produced several distinct patterns of risk perception and health-related behavior. Men with venous disease appeared most likely to trivialize symptoms and postpone medical consultations, often interpreting discomfort as an unavoidable aspect of aging. Women with arterial disease, by contrast, frequently displayed the highest levels of vigilance and anxiety, reflecting the perceived severity of possible complications combined with a heightened attentiveness to bodily signals.

Other groups occupied intermediate positions within this spectrum. Women living with venous disease often engaged in regular symptom monitoring but framed their concerns primarily in terms of comfort, mobility, or appearance rather than serious medical risk. Men with arterial disease, meanwhile, tended to combine vigilant monitoring with a restrained emotional tone, acknowledging potential danger while emphasizing personal control over their condition.

These patterns suggest that both disease characteristics and gendered expectations shape how individuals interpret bodily symptoms and respond to medical risk. Sociological research has consistently shown that health behaviors are embedded within broader cultural frameworks that define appropriate ways of experiencing and expressing illness (Bury, 1982; Butler, 1999; Connell, 2005). Within these frameworks, gender influences not only how individuals understand their bodies but also how they interact with healthcare systems.

Importantly, these gendered interpretations also affected patterns of healthcare utilization. Women were generally more likely to seek medical advice or discuss symptoms with healthcare professionals, particularly when they perceived potential risks to their health or daily functioning. Men, in contrast, often delayed consultations until symptoms became more severe or disruptive to their routines. These differences did not necessarily reflect a lack of concern among male patients but rather the influence of cultural norms that encourage self-reliance and discourage frequent medical consultation.

These findings demonstrate that perceptions of vascular risk are shaped through the interaction between disease type and gendered social expectations. While arterial disease tends to produce heightened vigilance and anticipatory anxiety, venous disease is more often normalized and integrated into everyday life. Gender further modifies these patterns by influencing how individuals attend to their bodies, express concern, and engage with healthcare services.

Recognizing these intersections is essential for understanding patient behavior within vascular care pathways. Healthcare professionals may interpret delayed consultations or minimized symptoms as signs of indifference or non-compliance, when in fact they often reflect culturally embedded expectations about masculinity, aging, and bodily resilience. Similarly, heightened vigilance among some patients may be understood not simply as anxiety but as a rational response to perceived risk shaped by social and cultural meanings of disease.

By examining these gendered interpretations, this study highlights the complex ways in which vascular illness is experienced and managed in everyday life. Patients' responses to symptoms are not determined solely by biomedical factors but are mediated by social norms, cultural narratives, and personal biographies. Understanding these dynamics can contribute to more nuanced approaches to patient communication and care, recognizing that perceptions of risk and responsibility are deeply embedded within broader social contexts.

Although these gendered patterns were recurrent across the interviews, they were not uniform or deterministic. Not all men minimized symptoms or delayed healthcare seeking, and not all women described heightened bodily vigilance or concern. Rather, gender appeared to operate as one dimension within a more complex configuration of individual and social influences. Participants' responses also varied according to age, previous experiences of illness, familiarity with vascular disease among relatives or acquaintances, occupational and family responsibilities, perceived symptom severity, and previous encounters with healthcare services. In some accounts, these factors appeared to reinforce culturally gendered expectations, whereas in others they attenuated them. The observed differences should therefore be interpreted as recurring gendered tendencies within the sample rather than as fixed characteristics of men and women. This variability underscores the importance of understanding gender as relational and context-dependent, intersecting with individual biography, social roles, and disease experience in shaping perceptions of vascular risk and healthcare behavior.

## 5 Discussion

In this article, we have examined patients' perceptions of vascular risk through a sociological lens, highlighting how risk is socially constructed, gendered, and embodied rather than simply medically defined. Our findings reveal that arterial patients consistently perceive their condition as high risk, which translates into heightened anxiety, constant monitoring of bodily signals, and anticipatory behaviors aimed at preventing catastrophic events such as heart attacks, strokes, or amputations.

In contrast, venous patients often interpret their condition as minor, normalizing symptoms and prioritizing comfort or aesthetic concerns. This divergence illustrates that risk perception is not uniform across disease types but is shaped by broader social hierarchies of illness, where the biomedical severity of arterial disease confers higher social and psychological urgency compared to the more socially trivialized venous disease. The marked differences observed between arterial and venous patients suggest the need for a vascular sociology capable of analyzing vascular disease not only as a biomedical condition, but as a socially stratified, culturally interpreted, and embodied experience of risk (Costa and Serra, 2026a).

From the perspective of risk society theory (Beck, 1992; Giddens, 1991), these findings suggest that contemporary patients live in a context where health risks are both individualized and moralized. Arterial patients' constant attention to changes in their bodies demonstrates the internalization of systemic uncertainties about potentially life-threatening outcomes, producing a form of embodied hyper-vigilance. Venous patients' minimization, in contrast, reflects cultural and social practices that allow individuals to negotiate risk, prioritizing everyday functioning and coping over intensive preventive engagement (Douglas, 1992; Lupton, 2013). In this sense, vascular risk is both a cultural and corporeal experience, constructed through social discourse, biomedical knowledge, and personal experience.

Gendered patterns further nuance these perceptions. Male patients, particularly those with venous disease, often downplay symptoms, delay medical consultations, and emphasize stoicism, reflecting culturally reinforced norms of masculinity that valorize self-reliance and endurance (Connell, 2005; Courtenay, 2000). Women, by contrast, demonstrate heightened vigilance, particularly when managing arterial conditions or when venous disease affects functionality or appearance. These gendered vascular risk narratives reveal that men and women interpret, communicate, and act upon health risk in socially and culturally differentiated ways. Men's narratives often frame vigilance as rational and controlled, with minimal emotional engagement, whereas women's narratives foreground bodily awareness, emotional anticipation, and the impact of disease on social and aesthetic dimensions.

The intersection of gender and disease type also illustrates the interplay between social norms and individual experience. Men with venous disease were the most likely to trivialize risk and postpone care, whereas women with arterial disease exhibited intense embodied anxiety and persistent monitoring. Women with venous disease were attentive primarily to comfort and appearance, while arterial men demonstrated precautionary vigilance but often suppressed emotional expression. These patterns underscore that risk perception is both socially mediated and biologically experienced, with significant implications for patient engagement, preventive behavior, and health outcomes.

Importantly, the gendered patterns identified in this study should not be interpreted as universal or binary behavioral profiles. Considerable within-gender variation was evident, suggesting that gender interacts with age, individual biography, previous illness experiences, social roles, and encounters with healthcare in shaping vascular risk perception. Gender therefore represents a socially situated influence on illness behavior rather than a deterministic predictor of how individual men or women will perceive symptoms or engage with care.

Our findings align with scholarship on embodiment and chronic illness (Bury, 1982; Corbin and Strauss, 1985; Mol, 2008), demonstrating that risk is materially and affectively lived. The anticipatory behaviors of arterial patients exemplify the integration of cognitive, emotional, and bodily dimensions in risk perception, whereas venous patients' normalization strategies illustrate how social and cultural constructions of risk can mitigate embodied anxiety. These observations reinforce the notion that illness experiences and health behaviors cannot be fully understood without attending to both cultural meanings of risk and the embodied lived experience of disease.

Moreover, the concept of vascular risk hierarchies emphasizes that social valuation of disease severity shapes patient behavior. Arterial disease occupies a “high-risk” position that demands vigilance and proactive engagement, while venous disease is socially trivialized, often delaying care-seeking. These hierarchies are not merely biomedical but reflect broader social and cultural understandings of which illnesses are urgent and which can be managed passively or normalized. Recognizing these hierarchies is crucial for understanding disparities in preventive care, healthcare utilization, and patient anxiety.

Finally, our study highlights the importance of narrative approaches in understanding risk. Men and women construct and communicate their experiences of vascular risk differently, reflecting both social norms and embodied experience. Attending to these gendered vascular risk narratives allows healthcare providers to better understand patients' decision-making, anxieties, and preventive behaviors, and to develop strategies that are responsive to both social context and lived experience.

### 5.1 . Strengths and limitations

This study has several strengths, including the direct comparison of patients living with arterial and venous disease and the integration of risk perception, gender, and embodied illness experience within a qualitative sociological framework. Nevertheless, several limitations should be acknowledged. First, the study involved a relatively small sample of 28 participants recruited within a specific geographical and healthcare context in Calabria, Southern Italy. Although the sample size is consistent with the exploratory and interpretive aims of qualitative research, the findings are intended to provide contextualized and theoretically informed interpretations rather than statistical generalization. Their transferability to other populations and settings should therefore be considered with caution. Cultural understandings of vascular disease and gender, as well as healthcare accessibility, organization of care, and patient–professional communication, may differ across Italian regions and healthcare systems and may influence how vascular risk is perceived and experienced. At the same time, the detailed characterization of the study context and participants may allow readers to assess the relevance and potential transferability of the findings to comparable settings.

Second, although the analysis focused primarily on disease type and gender, other individual and social characteristics may have contributed to variation in participants' experiences. The sample included individuals across a relatively broad age range, and factors such as age, socioeconomic position, occupation, family responsibilities, previous experiences of illness, disease duration and severity, and familiarity with vascular disease may intersect with gender in shaping symptom interpretation, risk perception, and healthcare-seeking behavior. These dimensions were not systematically examined as independent analytical categories in the present study and should be explored more explicitly in future research.

Finally, the findings are based on participants' retrospective self-reported accounts and should be understood as narratives of illness experience rather than direct measures of actual health behavior or objective vascular risk. Similarly, the gendered patterns identified in the analysis represent recurring tendencies within this sample and should not be interpreted as universal or deterministic differences between men and women. Future research involving more socially and geographically diverse samples, different healthcare contexts, and longitudinal designs could examine the extent to which these patterns persist across settings and explore more systematically the intersections among gender, age, socioeconomic position, disease characteristics, and healthcare organization.

## 6 Conclusion

In conclusion, our analysis shows that vascular risk perception is the product of complex interactions between disease type, gendered social norms, and embodied experience. By situating these findings within broader sociological frameworks of risk society, cultural constructions of risk, and embodiment, we provide new insights into how patients interpret, respond to, and communicate health risk. These insights have important implications for patient-centered care, preventive health strategies, and public health communication, highlighting the need to consider both the social and corporeal dimensions of risk in designing interventions and health policies.

## Data Availability

The original contributions presented in the study are included in the article/supplementary material, further inquiries can be directed to the corresponding author/s.
